# Compensation-Based Full-Filed Thermal Homogenization for Contrast Enhancement in Long Pulse Thermographic Imaging

**DOI:** 10.3390/s25071969

**Published:** 2025-03-21

**Authors:** Yoonjae Chung, Chunyoung Kim, Seongmin Kang, Wontae Kim, Hyunkyu Suh

**Affiliations:** 1 Eco-Sustainable Energy Research Institute, Kongju National University, 1223-24 Cheonan-daero, Seobuk-gu, Cheonan-si 31080, Republic of Korea; dbswosla79@kongju.ac.kr; 2 enesG Co., Ltd. 8, Techno 10-ro, Yuseong-gu, Daejeon 34026, Republic of Korea; chunyoung.kim@enesg.co.kr (C.K.); seongmin.kang@enesg.co.kr (S.K.); 3 Division of Mechanical and Automotive Engineering, Kongju National University, 1223-24 Cheonan-daero, Seobuk-gu, Cheonan-si 31080, Republic of Korea

**Keywords:** non-destructive testing, active thermographic testing, long pulse thermography, compensation method, thermographic image, defect detectability

## Abstract

Non-destructive testing (NDT) plays a crucial role in ensuring the structural integrity and safety of industrial facilities and components. Long pulse thermography (LPT), a form of active thermographic testing (ATT), has gained attention for its ability to detect subsurface defects efficiently. However, non-uniform thermal excitation and environmental noise often degrade the accuracy of defect detection. This study proposes an advanced thermographic inspection technique incorporating a halogen array (HA) lamp and a compensation methodology to enhance the reliability of defect detection. Two compensation methods, namely absolute temperature compensation (ATC) and temperature rate compensation (TRC), were developed to correct non-uniform thermal loads and improve the defect contrast. Experimental validation was conducted on A-type and B-type mock-up specimens with artificial subsurface defects (10–90% depth). The results demonstrated a significant enhancement in the signal-to-noise ratio (SNR), reaching up to a 42 dB improvement in severe defects. Furthermore, a quantitative evaluation method was proposed using SNR-based defect depth estimation models, improving the accuracy of defect sizing. This approach eliminates the need for complex amplitude and phase transformations, enabling direct defect assessment from temperature thermograms.

## 1. Introduction

Non-destructive testing (NDT) has become an indispensable tool for evaluating the structural integrity of various industrial components without causing material damage. Among the numerous NDT methodologies, thermographic testing (TT) has gained widespread recognition due to its non-contact nature, ability to inspect large areas rapidly, and effectiveness in detecting subsurface defects [1,2,3]. Within TT, long pulse thermography (LPT), a subcategory of active thermographic testing (ATT), has demonstrated significant potential in identifying and characterizing subsurface defects by utilizing controlled thermal excitation combined with thermographic imaging [4,5,6].

Subsurface defect detection is a critical aspect of ensuring the reliability and safety of the structures and components used in aerospace, manufacturing, nuclear power plants, and civil infrastructure. Undetected subsurface defects such as corrosion-induced thinning, delamination, and voids can compromise structural integrity, leading to catastrophic failures [7]. Conventional NDT methods, including ultrasonic testing (UT), radiographic testing (RT), and eddy current testing (ECT), have been extensively utilized for subsurface defect detection [8,9]. However, these methods exhibit inherent limitations, such as high equipment costs, lengthy inspection times, and susceptibility to material composition and environmental factors [10]. Conversely, LPT provides a fast, intuitive, and efficient means of assessing subsurface defects by leveraging thermal contrast variations between defective and non-defective regions [4].

Despite its numerous advantages, LPT suffers from certain drawbacks that hinder its widespread industrial application. A major challenge associated with LPT is non-uniform thermal excitation, which results in spatially varying temperature distributions across the inspected surface [11]. These temperature variations arise due to differences in the material properties, inconsistencies in the applied thermal load, and environmental interferences, leading to decreased contrast and a reduced signal-to-noise ratio (SNR). Additionally, external noise sources, including ambient reflections and uneven heating profiles, further degrade the detectability of defects and hinder the accuracy of defect quantification.

To address these challenges, various signal-processing techniques have been proposed to enhance defect detection in LPT [4,12,13,14,15]. Methods such as fast Fourier transform (FFT)-based phase transformation, pulse phase thermography (PPT), principal component thermography (PCT), and thermographic signal reconstruction (TSR) have been widely studied [13,16,17,18,19]. While these techniques improve defect contrast by filtering noise and extracting relevant thermal features, they often require extensive computational resources, making real-time defect assessment difficult. Furthermore, phase and amplitude transformations introduce additional complexities, which may not be suitable for practical industrial applications that demand fast and efficient defect evaluation.

In this study, we propose an advanced compensation methodology that enhances the detectability of defects in LPT by mitigating non-uniform heating effects. Two novel compensation techniques, namely absolute temperature compensation (ATC) and temperature rate compensation (TRC), have been developed to correct thermal inhomogeneities and improve SNR without complex post-processing. Unlike traditional signal-processing methods, the proposed approach enables direct defect quantification from temperature thermograms, significantly simplifying the inspection workflow. By systematically compensating for non-uniform heating, the accuracy of defect detection is improved, enhancing the applicability of LPT in real-world scenarios.

## 2. Theory

### 2.1. Active Thermographic Testing (ATT)

An ATT is applied in cases where the test object does not exhibit the self-emission or absorption of heat, or when a controllable external heat source is utilized to enable inspection without relying on the intrinsic thermal distribution of the object. This approach allows for the detection of relatively small or specific types of defects using various principles and forms of external heat sources. Additionally, when a quantitative evaluation of the defect size is required, ATT is employed by utilizing a controllable external heat source.

ATT techniques are categorized based on the type of heat energy source applied, including optical thermographic testing, vibro-thermographic testing, ultrasonic thermographic testing, eddy currents thermographic testing, and microwave thermographic testing. Among these, optical thermographic testing is widely studied due to its facile handling and control, low cost, and non-contact nature, making it applicable to various fields. Furthermore, optical thermographic testing techniques can be further classified according to the control method of the optical heat source, including lock-in thermography (LIT), long pulse thermography (LPT), pulsed thermography (PT), and step heating thermography (SHT) [3,10].

### 2.2. Long Pulse Thermogrpahy (LPT)

**Figure 1:**
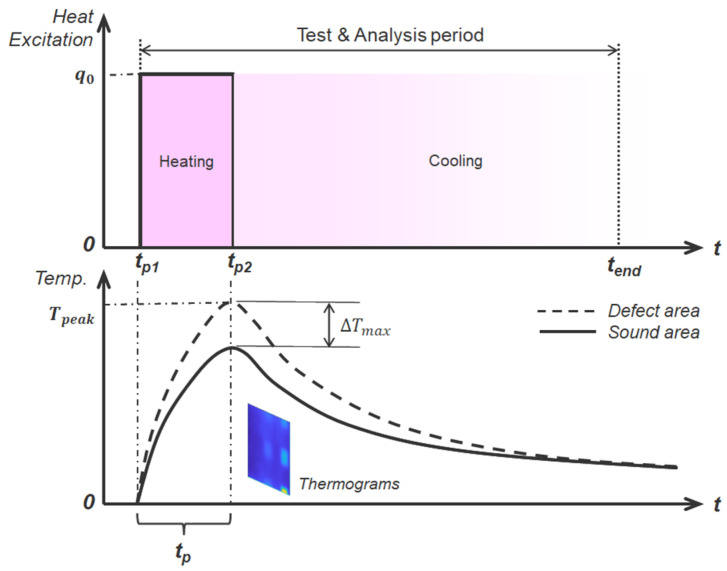
Principle of long pulse thermography (LPT).

LPT is a technique that delivers thermal excitation energy in the form of a strong pulse using electrical or optical heat sources. Figure 1 provides a detailed illustration of the surface temperature variation in LPT when a square pulse thermal load is applied. It distinctly represents the heating and cooling phases, differentiating the thermal response based on the presence or absence of defects in the test specimen. To ensure sufficient heat diffusion within the material and to measure the thermal response effectively, LPT applies a thermal pulse over a relatively extended duration, ranging from several seconds to several minutes. The prolonged heat pulse allows thermal energy to penetrate deeper into the object, making it more effective for detecting deep-seated or internal defects, as well as capturing temperature variations within the internal structure. Defects located at greater depths are more distinguishable at lower frequencies, whereas shallow defects are more readily detected at higher frequencies. However, the data acquired through long-duration pulses are more susceptible to environmental influences, which may require additional information to accurately determine the defect’s location and size. Consequently, LPT exhibits increased sensitivity to non-uniform thermal excitation, surface emissivity variations, and system noise, potentially imposing constraints on the practical implementation of inspections and defect evaluations [20,21].

The fundamental principle of LPT is governed by the heat conduction equation, which describes the diffusion of thermal energy within a material. The heat conduction equation in its three-dimensional form can be defined by Equation (1).

(1) $$\frac{\partial T}{\partial t}=\alpha \nabla^{2}T$$

where T is the temperature field, ∇ represents the spatial rate of change in a scalar field, t represents time, α is the thermal diffusivity, defined as $\alpha =\frac{k}{\rho c_{p}}$, k is the thermal conductivity, ρ is the material density, and $c_{p}$ is the specific heat capacity.

The LPT applies a thermal pulse for a prolonged period ($t_{p}=t_{p2}$ − $t_{p1}$) to ensure adequate heat diffusion into the material. The surface temperature as a function of time during heating can be expressed by Equation (2).

(2) $$T\left(t\right)=\frac{q_{0}}{\rho c_{p}}\left(t_{p}-\frac{t}{2}\right)\;\;\;\;\;for\;\;\;\;\;t_{p1}<t\leq t_{p2}$$

where $q_{0}$ represents the heat flux applied to the material, $t_{p1}$ is the heating start time, $t_{p2}$ is the heating end time, and $t_{end}$ is the experiment end time. In general, $T_{peak}$ is the temperature at the end of heating, and when a thinning defect exists, the temperature difference between the sound and the defect represents the maximum temperature difference (${\Delta T}_{max}$).

Following the heating phase (t > $t_{p}$), the cooling behavior of the surface temperature can be approximated by Equation (3).

(3) $$T\left(t\right)\approx \frac{q_{0}}{\rho c_{p}}\left(\frac{t_{p}}{2}\right)exp\left(-\frac{\pi^{2}\alpha \left(t-t_{p}\right)}{4d^{2}}\right)\;\;\;\;\;for\;\;\;\;\;t_{p2}<t<t_{end}$$

where *d* denotes the depth of the defect. This equation indicates that the surface temperature decreases exponentially with time after the application of heat, and the rate of temperature decay is influenced by the depth of subsurface defects.

### 2.3. Equipment Calibration and Compensation

#### 2.3.1. IR Camera Characteristics and Calibration

In order to apply thermographic testing NDT, accurate and stable temperature measurement, as well as precise, undistorted imaging and signal processing, are required. Currently, most commercially available infrared (IR) cameras incorporate a precisely fabricated detector based on a two-dimensional focal plane array (FPA) with pixel resolutions of 320 × 240 to 640 × 480. The size, resolution, and sensitivity of individual pixels in the FPA significantly influence the performance of IR cameras and the quality of thermal images. However, due to the detector’s arrangement and the non-uniform sensitivity of individual pixels, various non-uniformity correction (NUC) techniques have been developed. Generally, NUC is performed by deriving and applying offset correction values through an algorithm based on acquired sample images, thereby generating a uniform thermal image. This process typically considers the mean image of the acquired frames.

As a result of NUC in infrared cameras, the output value ($Y_{x,y}$) at an arbitrary pixel position (x, y) in the detector can be expressed as a linear Equation (4) with respect to the surface temperature (T), considering the gain ($G_{x,y}$) and offset ($O_{x,y}$) corrections for each pixel [22].

(4) $$Y_{x,y}=G_{x,y}\times T+O_{x,y}$$

#### 2.3.2. Compensation Method of IR Image

The inspection device applied to ATT may experience non-uniform thermal loads due to factors such as the position and irradiation direction of the light source, variations in the uniformity of the lamp output, and the shape of the reflective surface. These non-uniform thermal loads can cause variations in the thermal response of the inspection target’s surface, leading to unintended contrast differences in the measured thermographic images. Furthermore, if external sunlight or surface reflections from the light source enter the camera, they may create intense hot spots on the thermal image. This can degrade the image quality and potentially affect the detection of defects.

**Figure 2:**
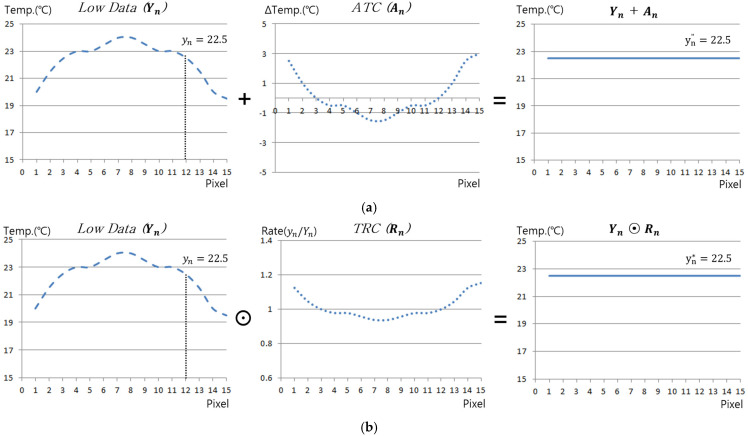
Principle of thermographic compensation methods: (**a**) ATC, (**b**) TRC.

In this study, to improve the accuracy and defect detectability of LPT, the thermal effects and responses of halogen lamps and ambient light sources that may arise during the inspection process were pre-evaluated using a reference specimen. During this process, a compensation sheet was generated by adjusting all pixel values in each thermographic frame relative to a reference pixel, ensuring uniform correction across the entire image set. This compensation sheet was then applied to thermographic images obtained from actual inspections to improve unintended contrast variations and hot spot effects. In this study, two methods able to compensate for IR images were proposed. Figure 2 presents the principles and differences between the two approaches [23].

(1) Absolute Temperature Compensation (ATC)

The absolute temperature compensation (ATC) method compensates temperature values by ensuring that the temperature of other pixels is uniformly adjusted relative to a reference pixel’s temperature value. This compensation is applied in a matrix form to each thermographic frame, as expressed in Equation (5).

(5) $$A_{n,x,y}=y_{n}-Y_{n,x,y}$$

(2) Temperature Rate Compensation (TRC)

The temperature ratio compensation (TRC) method, on the other hand, compensates temperature values based on the ratio between a reference pixel’s temperature and the temperature of other pixels, ensuring a uniform adjustment. Similar to ATC, TRC is also structured in a matrix form, as expressed in Equation (6).

(6) $$R_{n,x,y}=\frac{y_{n}}{Y_{n,x,y}}$$

where $Y_{n,x,y}$ represents the measured temperature value at position (x, y) in the nth thermographic image, while $y_{n}$ denotes the temperature value of the reference pixel in the nth thermographic image. $A_{n,x,y}$ and $R_{n,x,y}$ correspond to ATC and TRC, respectively, which uniformly adjust the entire nth thermographic image relative to the reference pixel’s value.

The ATC and TRC models, generated using a reference specimen, can improve the effects of non-uniform irradiation from sources such as lamps during actual inspections conducted under the same testing conditions and with the same equipment. Consequently, the compensation sheets can be applied to all thermographic images acquired during the actual inspection, as formulated in Equations (7) and (8).

(7) $$Y_{n,x,y}^{\prime \prime }=Y_{n,x,y}^{{}^{\circ}}+A_{n,x,y}$$

(8) $$Y_{n,x,y}^{*}=Y_{n,x,y}^{{}^{\circ}}⊙R_{n,x,y}$$

where ⊙ is a symbol representing the Hadamard product, $Y_{n,x,y}^{{}^{\circ}}$ represents the thermographic image acquired from the mock-up specimen, $Y_{n,x,y}^{\prime \prime }$ denotes the thermographic image with ATC applied, and $Y_{n,x,y}^{*}$ corresponds to the thermographic image with TRC applied.

## 3. Methods

### 3.1. Reference and Mock-Up Specimen

The test specimens considered in this study target subsurface defects caused by corrosion and are categorized into reference specimens and mock-up specimens based on their purpose and application. The test specimen is 6 mm thick, 300 mm × 300 mm in size, and is made of SS 275 metal material in the shape of a square plate.

**Figure 3:**
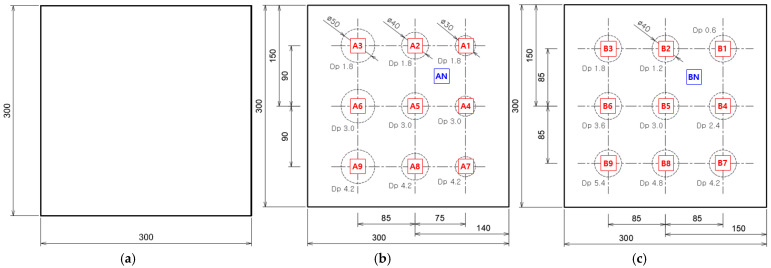
Geometric information of standard and mock-up sample: (**a**) Defect-free standard (STD) specimen, (**b**) A-type mock-up specimen, (**c**) B-type mock-up specimen.

Figure 3 illustrates the geometric details of the reference and the mock-up sample. Figure 3a illustrates the defect-free specimen, which was utilized to pre-assess the effects of non-uniform thermal heating and the temperature distribution variations induced by the heat source during the LPT testing. This specimen was subsequently used to generate a compensation sheet for thermal correction. In the mock-up specimen, artificial defects were arranged with a minimum spacing of 40 mm between each defect in both the row and column directions. The defects were uniformly distributed within the inspectable area, and based on the configuration of the included defects, the mock-up specimens were further categorized into A-type and B-type.

Figure 3b illustrates the A-type mock-up specimen, which was used in thermographic experiments to evaluate the detectable area corresponding to different thinning depths under the LPT inspection conditions. The A-type mock-up specimen contains a total of nine artificial defects, machined on the subsurface of the plate, where each row has defect depths of 30% (1.8 mm), 50% (3.0 mm), and 70% (4.2 mm), while each column has defect diameters of 30 mm, 40 mm, and 50 mm. Additionally, for defect detection and evaluation, a series of defect IDs were assigned, ranging from AN (sound region) to A9.

Figure 3c illustrates the B-type mock-up specimen, which was used in thermographic experiments to assess the detectability and depth resolution for a constant defect size. The B-type mock-up specimen consists of a total of nine artificial defects, each with a constant diameter of 40 mm on the subsurface, but with varying defect depths ranging from 10% (0.6 mm) to 90% (5.4 mm). Furthermore, to facilitate defect identification and evaluation, a series of defect IDs were assigned, ranging from BN (sound region) to B9.

### 3.2. Configuration of Test Equipment

**Figure 4:**
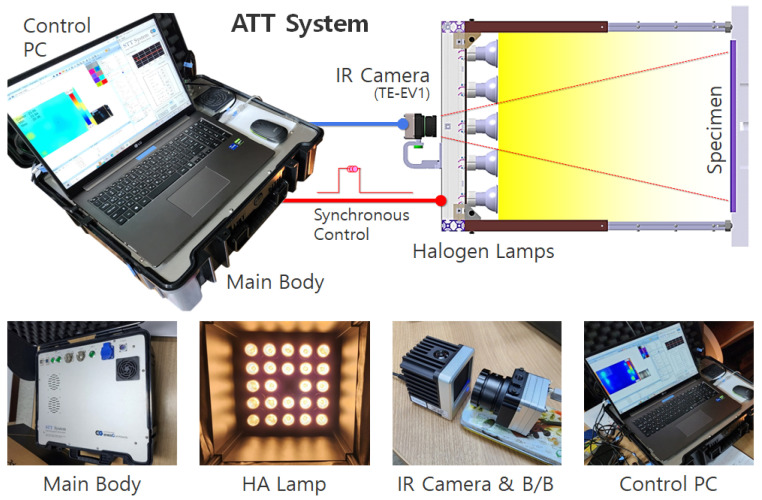
Configuration of the active thermography testing system.

Figure 4 presents the ATT system employed in this study. This system was designed with the objectives of automation, weight reduction, enhanced field applicability, and improved inspection accuracy. The ATT system comprises a main body housing the key electronic components, halogen lamps serving as the thermal excitation source, an IR camera for measurement, and a personal computer (PC) for signal control, data acquisition, and analysis.

The main body of the ATT system incorporates a function generator that controls the waveform and cycle for halogen lamp operation, a power amplifier that supplies and regulates power to the lamp based on the controlled signal, and a high-capacity storage unit for acquired thermographic data. All components within the system are designed to be centrally controlled via a dedicated PC.

For thermal excitation, the ATT system employs halogen lamps, which offer advantages in terms of easy procurement and maintenance. Instead of conventional large-capacity parabolic halogen lamps, which exhibit hotspot phenomena, a halogen array lamp (HA lamp) was specifically designed and developed to ensure uniform thermal energy distribution across the entire inspection area. The HA lamp features a central through hole for IR camera installation, with 24 halogen bulbs (50 W each) arranged in a 5 × 5 two-dimensional array, excluding the central position occupied by the IR camera. Additionally, the system is coated with matte black paint on the sidewalls and rear panel to minimize light reflection and external heat interference.

In this study, the TE-EV1 infrared thermographic camera from i3system Inc. (Daejeon, Republic of Korea) was integrated into the ATT system configuration. The TE-EV1 has a resolution of 640 × 480 pixels and a long-wave infrared range of 8 to 14 μm.

### 3.3. Analysis Process

**Figure 5:**
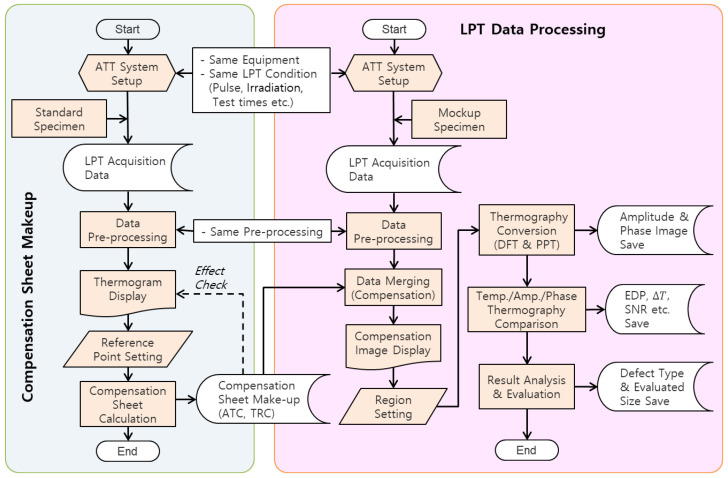
Flowchart of LPT data processing and analysis.

Figure 5 presents a comprehensive flowchart of the methodology employed in this study. The LPT experiment and subsequent analysis can be categorized into two main processes. The first step involves identifying surface temperature non-uniformities through LPT experiments on a standard specimen and deriving a compensation sheet. The second step involves utilizing the derived compensation sheet in experiments with a mock-up specimen. This is followed by systematic signal processing and quantitative analysis to accurately detect and assess defects. The detailed methodology and specific analytical procedures are outlined as follows: the generation and application of a compensation sheet for non-uniform thermal loading based on standard specimens.

(1) The pre-processing of raw transient temperature data acquired from the LPT inspection.
(2) The transformation of amplitude and phase images using fast Fourier transform (FFT) and pulse phase thermography (PPT) techniques.
(3) The post-processing of temperature, amplitude, and phase images for defect detection and evaluation, including comparative analysis.

**Figure 6:**
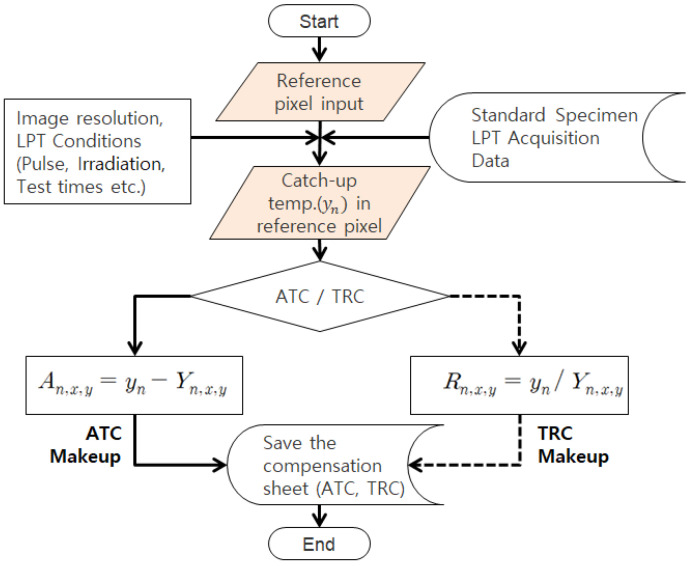
Flowchart of ATC and TRC compensation methods.

In this study, the HA lamp was developed and applied to improve the non-uniform thermal loading caused by external thermal excitation from lamps. Simultaneously, a method was considered to compensate for the non-uniform thermal heating present in the raw thermographic data through subsequent signal processing in analysis software, using MATLAB R2024b. To achieve this, a defect-free standard specimen was utilized in combination with the ATT system under identical conditions and procedures as the actual LPT testing. This setup allowed for the preliminary identification of the transient-state thermographic contrast and non-uniformities caused by the thermal excitation lamp. As shown in Figure 6, a compensation sheet was generated to adjust such contrasts across all frames and normalize the thermal distribution to a single, uniform value.

Initially, the inspector designates a reference pixel, and two distinct compensation sheets are formulated: (1) an ATC compensation sheet, which adjusts the temperature deviations of other pixels based on the reference pixel’s temperature value in each frame, and (2) a TRC compensation sheet, which calculates the temperature ratio between the reference pixel and other pixels, followed by inverse compensation to equalize the temperature distribution across frames. This method ensures the accurate derivation of the desired compensation sheet. The resulting compensation sheet can be applied to the actual LPT inspection data using matrix summation or multiplication methods.

#### 3.3.1. Pulsed Phase Thermography (PPT)

LPT is an NDT technique in which a long-term heat pulse is applied, rather than a short pulse, to induce thermal diffusion in the inspected material. If internal defects are present, the heat flow is disrupted, resulting in thermal anomalies that can be visually detected. Pulsed Phase Thermography (PPT) is a widely used non-destructive testing (NDT) technique that enables effective subsurface defect detection by analyzing thermal responses in the frequency domain. Unlike traditional thermographic methods that rely on direct temperature contrast, PPT enhances defect visibility by converting transient thermal signals into phase and amplitude components using Fourier Transform [24].

In the PPT technique, fast Fourier transform (FFT) is employed to analyze the thermal response data in the frequency domain. By applying FFT, the thermal signals measured in the time domain can be transformed into phase (∅) and amplitude (A) components, enabling more precise defect detection. The FFT is an algorithm designed to efficiently compute the discrete Fourier transform (DFT), which is mathematically represented as Equation (9).

(9) $$X\left(k\right)=\sum_{n=0}^{N-1}{x\left(n\right)e}^{-i2\pi kn/N},\;\;\;\;\;k=0,1,\cdots N-1$$

where $X\left(k\right)$ represents the transformed signal in the frequency domain, $x\left(n\right)$ is the sampled signal in the time domain, N denotes the total number of samples, k is the frequency index, and i is the imaginary unit ($i^{2}=-1$). The FFT algorithm accelerates the computation of the DFT, thereby enabling the efficient frequency domain analysis of thermal signals.

Following the FFT transformation, the amplitude $A\left(f\right)$ and phase $\emptyset \left(f\right)$ at a specific frequency f can be expressed as Equations (10) and (11), respectively [10].

(10) $$A\left(f\right)=\sqrt{{X(f)}^{2}+{Y(f)}^{2}}$$

(11) $$\emptyset \left(f\right)={tan}^{-1}\left(\frac{Y(f)}{X(f)}\right)$$

where X(f) and Y(f) represent the real and imaginary components of the transformed signal at frequency f, respectively. FFT analysis enables a quantitative comparison between defective and non-defective regions, facilitating the detection of deep internal defects. Additionally, it contributes to the enhancement of the signal-to-noise ratio, improving the reliability and accuracy of defect detection in LPT.

#### 3.3.2. Signal-to-Noise-Ratio (SNR)

The SNR is a term that represents the ratio of the target signal to background noise and serves as a suitable parameter for expressing the strength and accuracy of a measured signal. A higher SNR indicates a clearer acquired signal with reduced noise, whereas a lower SNR signifies the greater influence of noise, making signal identification more challenging and resulting in a lower image quality.

In previous studies across various fields [5,6], the SNR calculation formula, as expressed in Equation (12), has been applied to evaluate image signals and assess image quality.

(12) $$SNR=20log\left(\frac{P_{S}}{P_{N}}\right)$$

where $P_{N}$ represents the signal intensity of the background region in the sound area, including the temperature, amplitude, and phase values, and $P_{S}$ represents the signal intensity of the defect area.

#### 3.3.3. Median and Absolute Value Evaluation

The suitability and effectiveness of the inspection techniques and equipment used in NDT can be evaluated based on various criteria, including the defect detectability, resolution, accuracy and reproducibility of the defect sizing, and the quantitative assessment of the detected defects. In ATT, extensive research has been conducted to develop optimal inspection techniques and quantitative evaluation methods based on different materials, inspection conditions, and defect types. Previous studies have presented techniques for the quantitative assessment of the defect width, length, depth, or thickness by analyzing transient temperature differences, amplitude variations, and phase shifts [24,25].

Furthermore, the American Society for Non-destructive Testing (ASNT) has compiled methodologies in the Non-destructive Testing Handbook, where two approaches are introduced for determining the apparent size of defects from thermographic images obtained through ATT. The median evaluation method utilizes a mid-value threshold, calculated as half of the difference between the mean signal intensity of the defect area and the mean intensity of the background sound area, to determine the apparent defect size (length and width for defects). Alternatively, the absolute evaluation method employs a predefined threshold for the binarization or direct determination of the apparent defect size [26,27].

### 3.4. LPT Testing Condition

Under testing conditions, the LPT inspection system was configured using the ATT system. The distance between the IR camera (TE-EV1) and the specimen was set to approximately 550 mm. Considering the IR camera’s resolution (640 × 480 pixels), the spatial resolution, defined as the instantaneous field of view (IFOV), was evaluated to be 0.47 mm/pixel. The distance between the inner halogen bulbs of the HA lamp and the specimen was set to approximately 400 mm, ensuring proper execution of the LPT inspection for both the standard and mock-up specimens. The pulse waveform control of the thermal excitation, the operation of the halogen lamps, the control of the IR camera, and the storage of inspection data were all fully automated through the control PC. The heating time of the lamp was selected as the primary variable in the LPT experimental conditions for the standard specimen and the A-type and B-type mock-up specimens. The heating time was set to 10, 20, and 30 s, respectively. To ensure sufficient heat diffusion within the material, the cooling time after heating was set to 40, 80, and 120 s, which correspond to four times the heating duration. The detailed LPT test conditions for each specimen are summarized in Table 1.

This study confirms the detectability of plate thinning using the ATT system and HA lamp. It derives the effective detectable period (EDP) and optimal evaluation time (OET) by analyzing trends in the temperature, phase, amplitude images, and signal-to-noise ratio (SNR) under each LPT test condition. Additionally, it compares and analyzes the quality of thermograms and the defect detectability. Furthermore, the application of ATC and TRC compensation sheets is examined, focusing on the improvement in thermogram uniformity and detection. Lastly, the quantitative evaluation and error rate of defect detection are analyzed.

## 4. Results and Discussion

### 4.1. Comparison of Temperature Contrast

**Figure 7:**
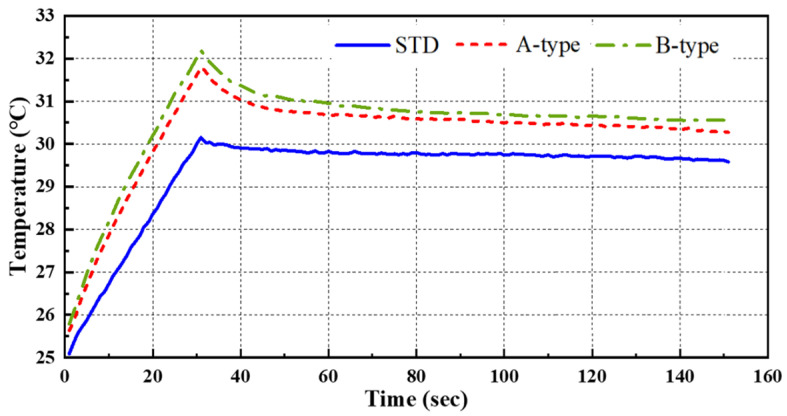
Temperature trends by LPT with HA lamp (@ 320,240 pixel of test #3).

**Figure 8:**
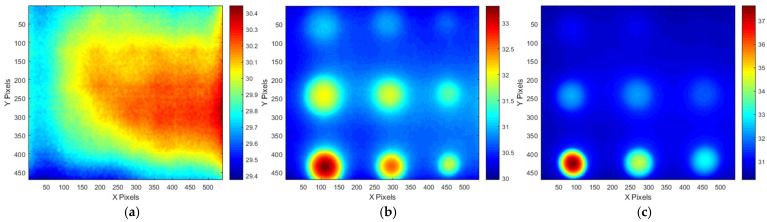
Temperature thermographic images by HA lamp (at #301 frame of test #3 LPT): (**a**) STD, (**b**) A-type, (**c**) B-type.

Figure 7 presents the transient temperature variation measured at the central pixel location (320,240 pixel) of the thermogram for STD and the A and B-type mock-ups under test #3 conditions, excluding the initial stabilization period. Figure 8 presents the temperature thermographic images at peak temperature, corresponding to the termination of thermal heating at frame #301, under test #3 conditions. As shown in Figure 7, all three specimens reached their peak temperature at frame #301 (30 s), corresponding to the termination of thermal heating; this was followed by a gradual decrease in temperature due to thermal diffusion and cooling. Additionally, as shown in Figure 8a, the standard specimen exhibited a temperature gradient ranging from 29.38 °C to 30.44 °C at the peak temperature frame, indicating a temperature difference of approximately 1.06 °C. Moreover, the temperature distribution in the sound region appeared relatively uniform across all thermal images. In contrast, Figure 8b,c demonstrate that the defect regions exhibited a significantly higher contrast compared to the sound regions, allowing for the identification of more than six distinct defect locations.

**Figure 9:**
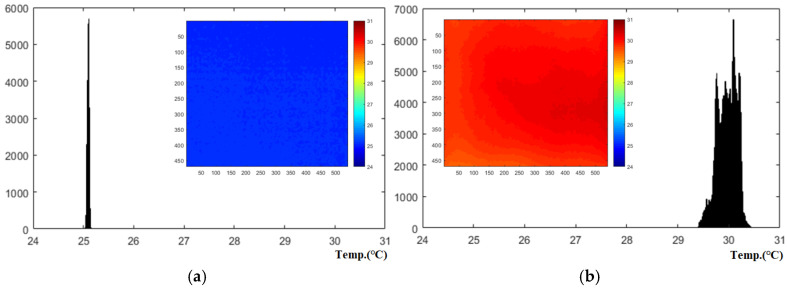
Histograms of temperature thermographic image of standard specimen (@test #3, temp., span: 24∼31 °C): (**a**) #1 frame, (**b**) #301 frame.

Figure 9 shows the LPT test results for the standard specimen in test #3, which has the highest heating intensity. It presents histograms for frame #1 (0 s, before heating) and frame #301 (30 s, peak temperature) within a temperature range of 24 °C to 31 °C. Table 2 provides the initial temperature, the temperature increase at the peak frame when lamp heating ends for each experimental condition, the average temperature, the maximum contrast based on the maximum and minimum temperatures, and the contrast ratio relative to the heated temperature. Additionally, the variance and standard deviation are presented to quantify the contrast uniformity of the peak frame under each heating condition.

Under the test #3 condition, the temperature increased by approximately 5.1 °C, from an initial temperature of 25.2 °C to 30.3 °C. The maximum contrast in the peak frame (#301) was around 1.1 °C. Across all experimental conditions, the surface temperature increase is generally proportional to the heating time, and the non-uniformity rate is estimated to be between 20% and 25% of the temperature increase.

### 4.2. Temperature Thermogarm and SNR Trend

**Figure 10:**
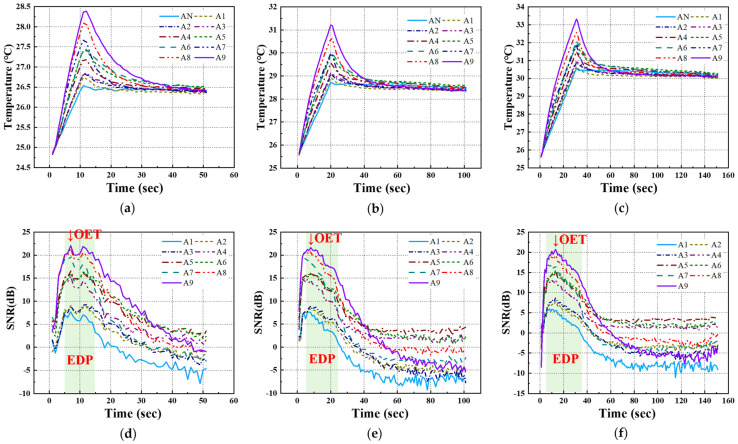
Temperature and SNR trend of A-type specimen by LPT: (**a**) temperature trend of test #1, (**b**) temperature trend of test #2, (**c**) temperature trend of test #3, (**d**) SNR trend of test #1, (**e**) SNR trend of test #2, (**f**) SNR trend of test #3.

**Figure 11:**
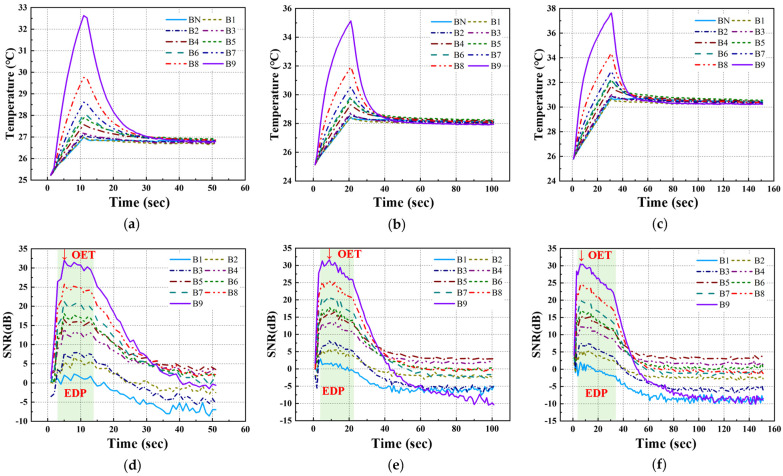
Temperature and SNR trend of B-type specimen by LPT: (**a**) temperature trend of test #1, (**b**) temperature trend of test #2, (**c**) temperature trend of test #3, (**d**) SNR trend of test #1, (**e**) SNR trend of test #2, (**f**) SNR trend of test #3.

For the temperature data acquired under each LPT testing condition for the A-type and B-type mock-up specimens, the transient temperature and SNR trends were analyzed, focusing on each defect ID region. Figure 10 and Figure 11 present the temperature and SNR trends under different LPT testing conditions for each mock-up specimen. It was observed that both A-type and B-type mock-up specimens exhibited a higher temperature gradient as the defect size increased and the depth increased.

In previous studies, the maximum SNR of the defect region was found to be ±12 dB or higher, and the period during which the SNR value exhibited a proportional relationship with defect size was defined as the EDP. During this period, the contrast between the sound and defect regions became significantly pronounced, facilitating the identification and detection of defects [5].

**Figure 12:**
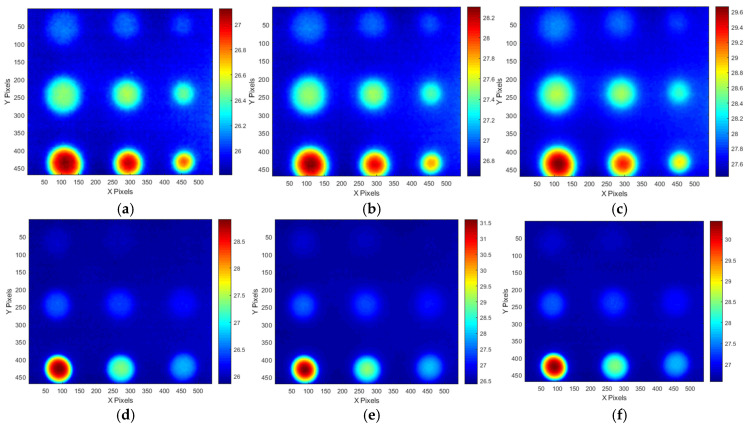
Temperature thermographic images of OET of each LPT testing condition: (**a**) #70 frame of A-type test #1, (**b**) #80 frame of A-type test #2, (**c**) #130 frame of A-type test #3, (**d**) #50 frame of B-type test #1, (**e**) #90 frame of B-type test #2, (**f**) #60 frame of B-type test #3.

Figure 12 shows temperature thermograms created at the OET for each LPT test condition. As shown in Table 3, the EDP and OET were determined based on the temperature contrast level and SNR trend for each testing condition. The application of the ATT system and HA lamp resulted in a detectability exceeding 30 dB for the B9 (Φ40 mm, 90% depth) defect, demonstrating excellent detectability across all testing conditions. However, for B1 (Φ40 mm, 10% depth) to B3 (Φ40 mm, 30% depth), which exhibited lower defect depths, the detection capability was relatively low, with SNR values below 12 dB. As a result, the temperature change was the highest in the Test #3 condition.

### 4.3. Amplitude and SNR Trend

**Figure 13:**
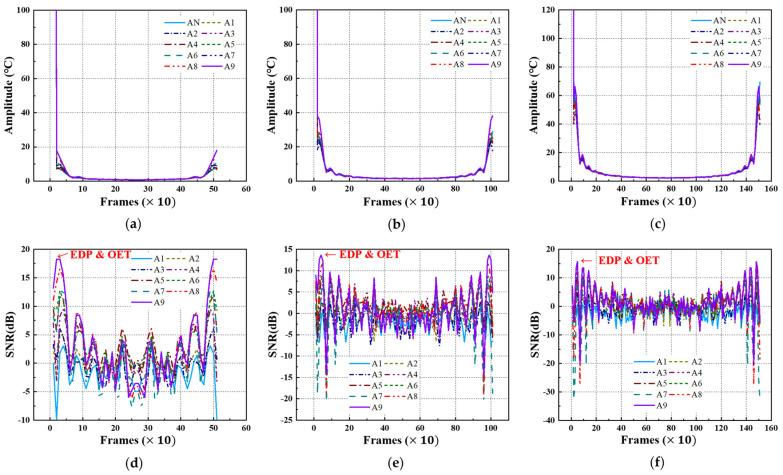
Amplitude and SNR trend of A-type specimen by LPT: (**a**) amplitude trend of test #1, (**b**) amplitude trend of test #2, (**c**) amplitude trend of test #3, (**d**) SNR trend of test #1, (**e**) SNR trend of test #2, (**f**) SNR trend of test #3.

**Figure 14:**
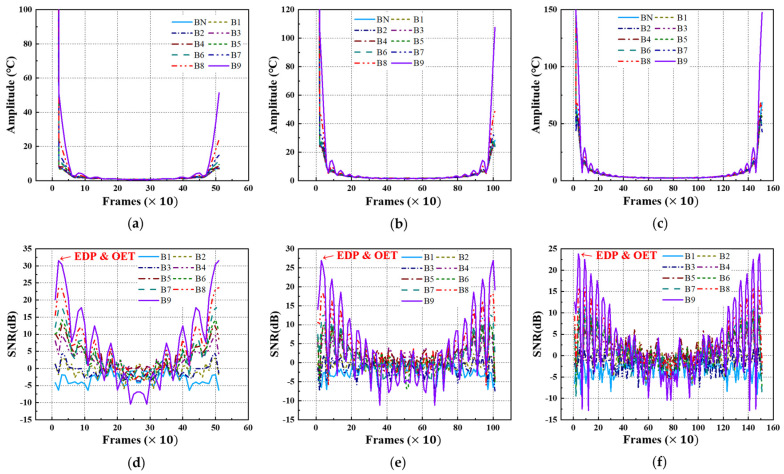
Amplitude and SNR trend of B-type specimen by LPT: (**a**) amplitude trend of test #1, (**b**) amplitude trend of test #2, (**c**) amplitude trend of test #3, (**d**) SNR trend of test #1, (**e**) SNR trend of test #2, (**f**) SNR trend of test #3.

Using FFT transformation, the acquired thermographic data for the A-type and B-type mock-up specimens under each LPT testing condition were converted into amplitude thermographic images. Figure 13 and Figure 14 present the transient amplitude values and SNR trends, focusing on each defect ID region in each specimen. As shown in Figure 13 and Figure 14, the transformation from the time domain to the frequency domain exhibits a pulsation pattern.

Table 4 presents the selected values of EDP and OET based on the amplitude values and SNR trends under each testing condition. Due to the pulsation phenomenon, the EDP was significantly narrowed, indicating that the evaluation timing must be carefully selected.

**Figure 15:**
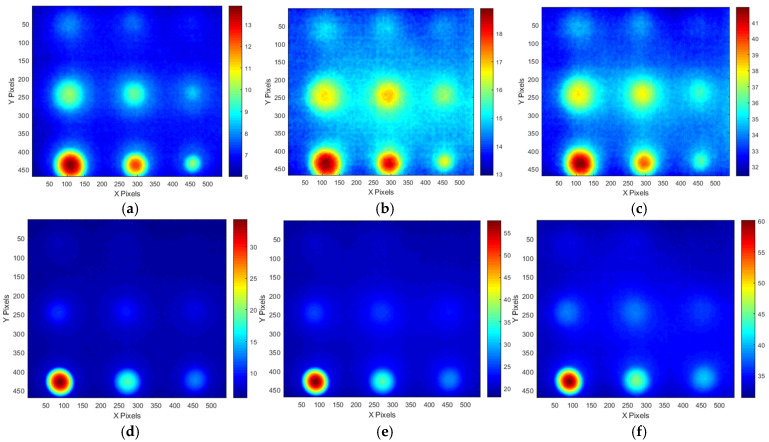
Amplitude images at OET of each LPT testing condition: (**a**) #30 frame of A-type test #1, (**b**) #50 frame of A-type test #2, (**c**) #50 frame of A-type test #3, (**d**) #30 frame of B-type test #1, (**e**) #40 frame of B-type test #2, (**f**) #50 frame of B-type test #3.

Upon examining the amplitude thermographic images corresponding to the selected OET for each testing condition, it was observed that the highest detectability, corresponding to B9 (Φ40 mm, 90% wall thinning), was approximately 20–30 dB. This indicates that, under all testing conditions, the quality and contrast of the amplitude thermographic images were inferior compared to the temperature-based thermographic images. Additionally, similar to the temperature-based thermographic images, the SNR was slightly better under test #1 condition, where the heating duration was relatively short. Figure 15 shows the amplitude images obtained at the OET for each LPT testing condition.

### 4.4. Phase and SNR Trend

**Figure 16:**
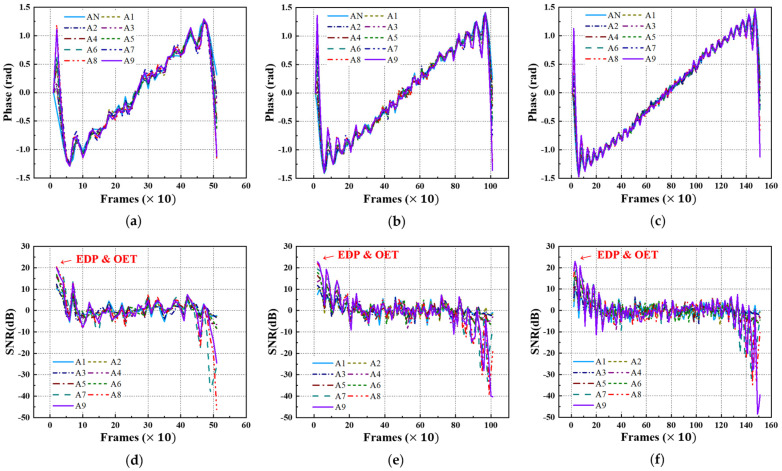
Phase and SNR trend of A-type specimen by LPT: (**a**) phase trend of test #1, (**b**) phase trend of test #2, (**c**) phase trend of test #3, (**d**) SNR trend of test #1, (**e**) SNR trend of test #2, (**f**) SNR trend of test #3.

**Figure 17:**
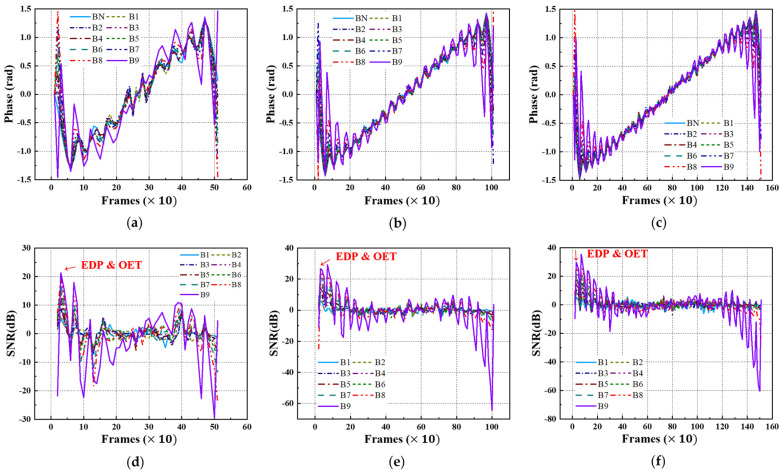
Phase and SNR trend of B-type specimen by LPT: (**a**) phase trend of test #1, (**b**) phase trend of test #2, (**c**) phase trend of test #3, (**d**) SNR trend of test #1, (**e**) SNR trend of test #2, (**f**) SNR trend of test #3.

Equivalently, using FFT transformation, the acquired thermographic data for the A-type and B-type mock-up specimens under each LPT testing condition were converted into phase thermographic images. Figure 16 and Figure 17 present the transient phase values and SNR trends, focusing on each defect ID region in each specimen. As shown in Figure 16 and Figure 17, similar to the amplitude results, the transformation from the time domain to the frequency domain exhibits a pulsation pattern.

Table 5 presents the selected values of the EDP and OET based on the phase values and SNR trends under each LPT testing condition. Similar to the amplitude results, the EDP was significantly narrowed due to the pulsation phenomenon, highlighting the need for the careful selection of the evaluation timing.

**Figure 18:**
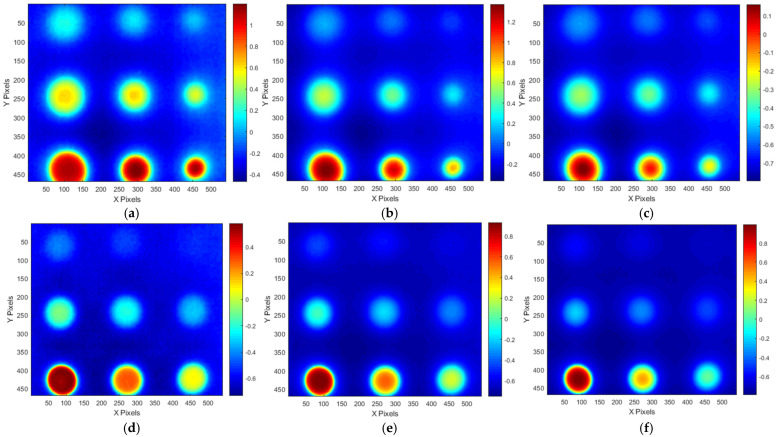
Phase images obtained at OET of each LPT testing condition: (**a**) #20 frame of A-type test #1, (**b**) #20 frame of A-type test #2, (**c**) #30 frame of A-type test #3, (**d**) #30 frame of B-type test #1, (**e**) #30 frame of B-type test #2, (**f**) #30 frame of B-type test #3.

Upon examining the phase thermographic images corresponding to the selected OET for each testing condition, it was observed that the highest detectability, corresponding to B9 (Φ40 mm, 90% depth), was approximately 20–30 dB, which was lower than that of temperature-based thermography. However, for specimens with lower defect depths, such as B1 (Φ40 mm, 10% depth) to B3 (Φ40 mm, 30% depth), the detectability was improved. In the phase thermographic image, the contrast of the thinned regions was slightly better under the Test #3 testing conditions compared to Test #1. Figure 18 presents the phase thermographic images for each OET.

### 4.5. Compensation Effect for Non-Uniform Heating

Thermographic testing data obtained under the conditions of test #3 for A-type and B-type mock-up specimens utilizing the ATT system and HA lamp were preprocessed by applying ATC and TRC compensation methods, derived from standard specimens.

**Figure 19:**
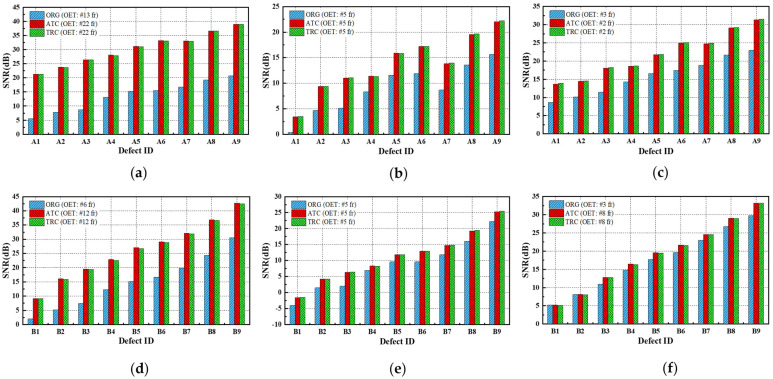
Comparison of SNR improvement effects by thermogram compensation of test #3: (**a**) A-temperature of A-type specimen, (**b**) amplitude of A-type specimen, (**c**) phase of A-type specimen, (**d**) temperature of B-type specimen, (**e**) amplitude B-type specimen, (**f**) phase of B-type specimen.

Figure 19 presents a comparative analysis of the detectability of the defect regions by evaluating the SNR between the original (ORG) acquisition data and the data processed with the ATC or TRC compensation methods. The effect of ATC or TRC compensation was evaluated more effectively in the temperature image than in the amplitude and phase to which the FFT transformation was applied, and the effect was evaluated to be higher for defects with smaller thinning defect amounts. In Figure 19a, the SNR of the A1 defect showed an SNR improvement effect of about four times from 5.5 dB to 21.3 dB by applying ATC or TRC compensation, and the SNR of A9 improved about two times from 20.7 dB to 38.9 dB. And in Figure 19d, the SNR of B1 improved about 2.5 times from 2.0 dB to 9.2 dB, and that of B9 improved about 1.4 times from 30.5 dB to 42.7 dB.

The compensation effect was similar for both ATC and TRC, and in the case of temperature images, the SNR improvement effect caused by ATC was generally slightly better. Therefore, the subsequent quantitative evaluation of the defects considered the temperature image with ATC applied.

**Figure 20:**
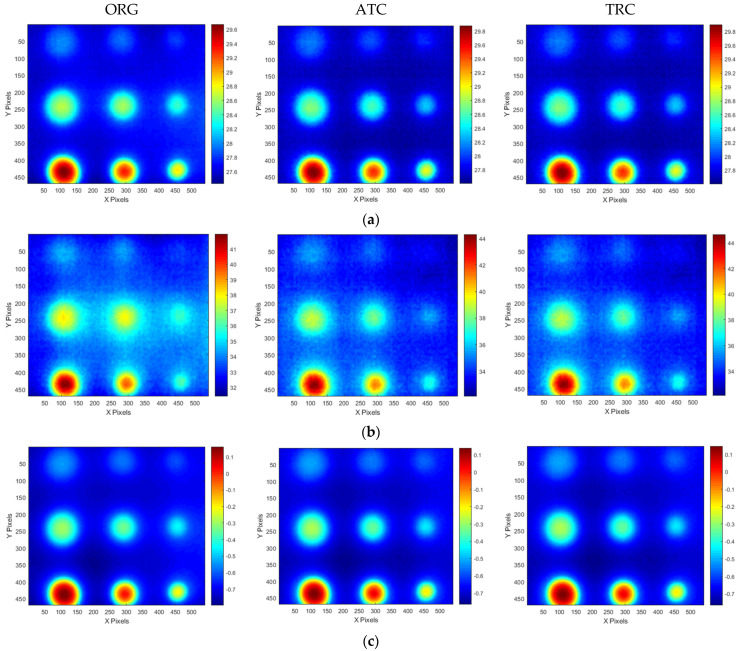
Thermographic image results for A-type mockup specimen test #3 at OET: (**a**) temperature, (**b**) amplitude, (**c**) phase.

**Figure 21:**
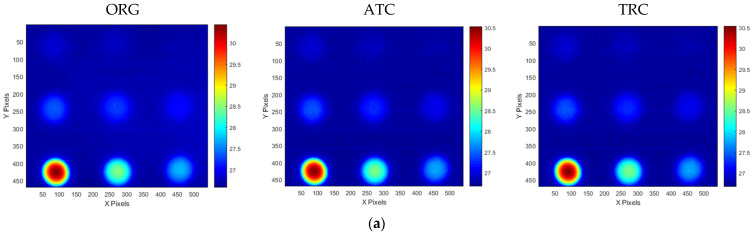

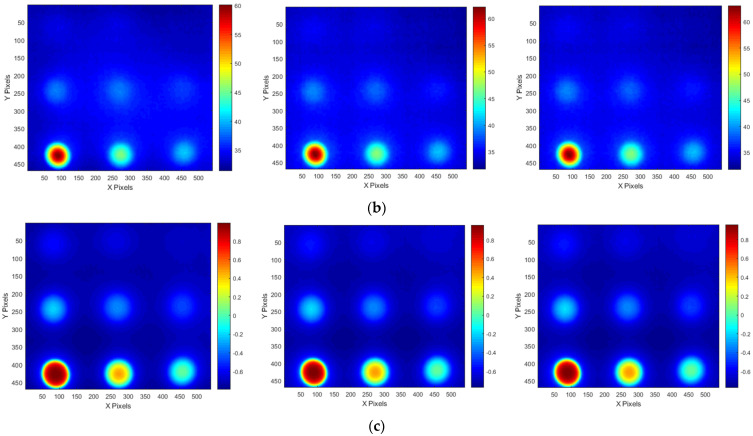
Thermographic image results for B-type mockup specimen test #3 at OET: (**a**) temperature, (**b**) amplitude, (**c**) phase.

Figure 20 and Figure 21 present the temperature, amplitude, and phase images corresponding to the OET for each specimen, depending on whether the compensation technique is applied. The results indicate that the application of the ATC or TRC compensation methods significantly enhances the SNR values of the temperature, amplitude, and phase images in the defect regions. In particular, the improvement in the SNR of the temperature thermograms was the most evident. For instance, the smallest wall-thinning defect, B1 (Φ40 mm, 10% depth), exhibited an SNR improvement exceeding 9 dB, whereas the most severe defect, B9 (Φ40 mm, 90% depth), exhibited an enhancement of over 42 dB. These findings suggest that the compensation process effectively improves the non-uniformity between the sound and surrounding areas in the temperature thermograms.

Furthermore, the application of the FFT algorithm, which is commonly used to address non-uniform thermal heat and noise, appears to interfere with the benefits of compensation. Therefore, when employing the ATT system, HA lamp, and LPT testing techniques in combination with non-uniform thermal heating compensation, it was found that an SNR improvement of over 1.4 times can be achieved without the need for complex post-processing, using only the temperature thermogram. Additionally, sub-surface defects with a thinning depth of 10% or more can be reliably detected.

### 4.6. Quantitative Evaluation of Defect Sizing and Depth

**Figure 22:**
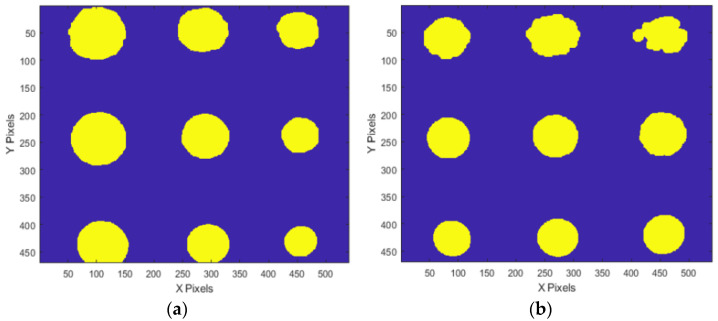
Binarized images of temperature thermogram by the median of each defect ID region: (**a**) A-type, (**b**) B-type.

Defect size evaluation was conducted using thermal images obtained at the OET of test #3. In this test, the ATC technique was applied due to its superior temperature uniformity correction. The median-based evaluation method was used for defect sizing assessment. Figure 22 presents the extraction of defect boundaries from the temperature thermograms of both mock-up specimens using binarization and morphological image processing, based on the median value of each defect ID region. The defect diameters were estimated by considering the pixel count of the binarized region in relation to the instantaneous field of view (IFOV).

**Figure 23:**
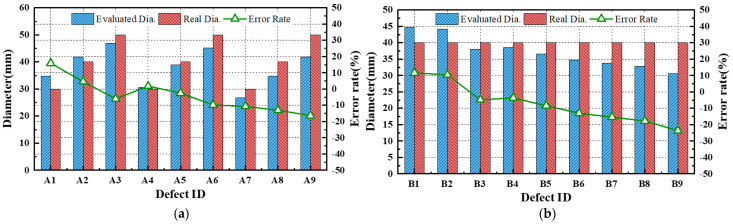
Comparison of actual and estimated diameters of defects: (**a**) A-type, (**b**) B-type.

Table 6 and Figure 23 present the estimated defect diameters obtained via the median-based evaluation, along with the actual diameters and percentage errors for each defect detected in both mock-up specimens. The results indicate that the diameter estimation error ranged from −24% to +16% for defects with depths between 10% and 90%. Additionally, smaller depth defects tended to be overestimated in terms of diameter. This phenomenon is likely attributed to the lower temperature gradient in regions with a minimal defect depth.

**Figure 24:**
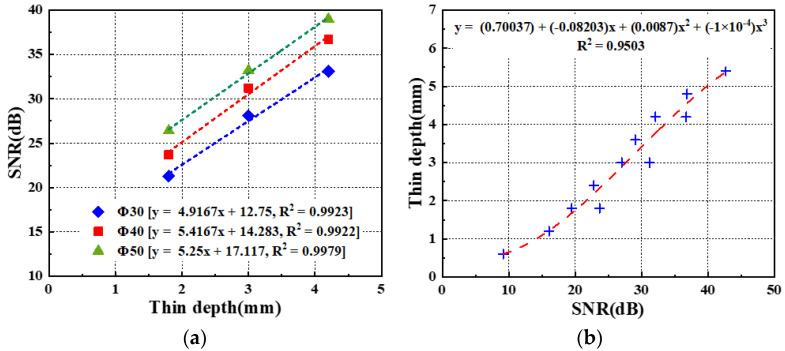
Relationship between the diameter, the depth, and the SNR of the defect area: (**a**) A-type, (**b**) All defects of Φ40 mm.

The evaluation of the depth in each defect ID region within the thermographic images selected using the OET was performed by establishing assessment criteria based on the SNR values obtained from mock-up specimen experiments under identical testing conditions. As shown in Table 6, the SNR values and defect sizes derived from the temperature thermograms of the OET, obtained through LPT test #3 with ATC applied, are presented for both A-type and B-type mock-up specimens. Figure 24a presents the relationship between the defect diameter and SNR for the A-type mock-up specimens. By analyzing the displacement of the intercept value as a function of the defect diameter, an empirical relationship between the defect diameter and SNR variation was derived, as expressed in Equation (13). Figure 24b presents the correlation between the defect depth and SNR for 12 defect regions with a diameter of Φ40 mm across both mock-up specimens. A polynomial fitting approach was applied to establish a correlation equation between the defect depth and SNR, as expressed in Equation (14). Subsequently, the two derived equations were integrated to formulate Equation (15), which defines the evaluation criteria for the defect depth based on the SNR values and the estimated defect diameters of the defect depth regions.

(13) ∆R=−0.2185d+8.3033

(14) $$t_{depth}=-0.0001R^{3}+0.0087R^{2}-0.082R+0.7004$$

(15) $$t_{depth}=-0.0001{\left(R+∆R\right)}^{3}+0.0087{\left(R+∆R\right)}^{2}-0.082\left(R+∆R\right)+0.7004$$

where $t_{depth}$ represents the defect depth on the subsurface (mm), R denotes the SNR of the defect region (dB), d refers to the diameter of the defect region (mm), and ∆R indicates the SNR correction value corresponding to the defect diameter (ΔdB).

In the case of evaluating the depth of a defect according to a relational formula, the smaller the thinning and the larger the area, the greater the evaluation error rate tends to be. This is presumed to be due to the effect of the edges of the thinning area, and it needs to be considered when evaluating the thinning thickness in actual inspections.

**Figure 25:**
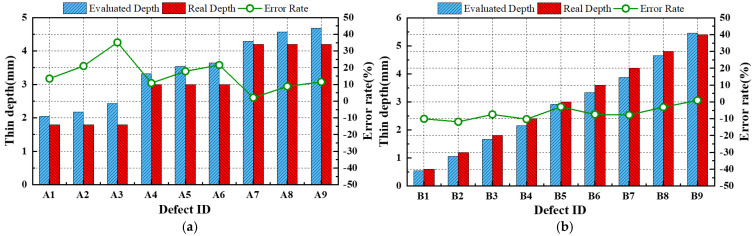
Comparison of the actual and estimated thin depth of defects: (**a**) A-type. (**b**) B-type.

The defect depths were evaluated by substituting the estimated defect diameters and SNR values from Table 6 into Equation (15). As shown in Table 6 and Figure 25, the error rates in the defect depth estimation ranged from −12% to +35% compared to the actual defect depths for both mock-up specimens.

The evaluated quantitative values exhibited two distinct characteristics. In the case of diameter evaluation using median-based assessment, the error rate tended to increase in the negative direction as the diameter increased or the defect depth became greater. This can be seen as a narrower median range as the SNR value of the defects is higher, and it is thought that this can be improved through additional compensation considering the SNR.

However, in deriving the relationship in this study, there is a slight imbalance of 1:3 between the A-type and B-type defect samples, which may introduce unintended bias into the quantitative evaluation of defects. To mitigate this potential bias in future studies, weighting adjustment factors could be applied to the regression model, or a more balanced sample could be considered to generalize the derived relationship. This approach could improve the error rate in defect assessment and should be considered for other material compositions or defect types.

To minimize the overestimation of the defect diameter, edge detection and region-based segmentation can be used to refine boundary identification. For shallow defects with low signal-to-noise ratio (SNR) values, post-processing techniques such as noise reduction and contrast enhancement can improve visibility. These methods will be further explored to enhance defect quantification. Additionally, FEM-based numerical models can complement LPT by simulating heat diffusion and defect interactions, leading to more accurate defect characterization. Integrating FEM analysis with experimental LPT results can improve the defect size and depth estimations, thereby reinforcing its value in thermographic defect evaluation. This approach has been investigated in previous studies, demonstrating its potential to enhance thermographic defect evaluation [28].

## 5. Conclusions

This study introduced ATC and TRC to enhance the accuracy of defect detection in long pulse thermography (LPT). The results demonstrated significant improvements in the thermal uniformity and defect contrast, effectively reducing thermal variations. In this study, the proposed ATT system, HA Lamp, non-uniform thermal heating compensation method, and evaluation methodology enabled the detection of a 10% to 90% defect depth in the subsurface, solely using temperature thermograms and thus eliminating the need for complex signal transformation processes.

The contributions of this study can be summarized as follows:

(1) The development and validation of a compensation-based thermal homogenization technique to correct non-uniform heating effects in LPT.
(2) The demonstration of significant improvements in SNR, improving detectability down to 10% thin defects.
(3) The introduction of an efficient, direct quantitative defect assessment method that eliminates the need for complex phase and amplitude transformations.
(4) Comprehensive experimental validation using realistic mock-up specimens, confirming the feasibility of our approach for industrial NDT applications.

Future work will focus on integrating spatial correction filters, such as Gaussian and Wiener filtering, to improve thermal homogenization. Additionally, adaptive correction models leveraging machine learning will be explored to dynamically adjust for localized variations in real time, further enhancing small defect detection. Moreover, to ensure the robustness of the proposed compensation techniques across different materials, future studies will expand the experimental framework to include composite and polymer-based materials. This will allow for a comprehensive assessment of how material-specific thermal properties influence defect detectability. Furthermore, additional strategies for minimizing environmental variations, such as real-time adaptive filtering and improved thermal load stabilization, will be investigated to enhance the reliability of defect detection in varying conditions. These findings contribute to the advancement of non-destructive testing by improving thermographic defect detection. The proposed compensation techniques enhance LPT’s reliability for industrial inspections, and future research will continue refining these methodologies for an optimal defect detection performance.

## Figures and Tables

**Table 1:** LPT experimental conditions for STD and mockup specimens.

Test No.	Initial Temp. (°C)	Time Composition (s)			Total Acquisition Frames ^(1)^	Frame Rate ^(2)^	IFOV
		Heating	Cooling	Total			
#1	25 ± 1	10	40	50	501	10	0.47
#2		20	80	100	1001		
#3		30	120	150	1501		

Note: ^(1)^ Thermographic image derived from heating start time to cooling end time. ^(2)^ Data acquisition speed of LPT experiment (unit: fps).

**Table 2:** Comparison of temperature contrast at peak frame by each LPT condition.

Test No.	Initial Temp. (°C)	Increase Temp. (°C)	Aver. Temp. (°C)	Min Temp. (°C)	Max Temp. (°C)	Max. Contrast (°C)	Un-Uni. Rate (%)	Variance $(\sigma^{2}$)	Standard Deviation (σ)
#1	24.7	1.8	26.5	26.2	26.7	0.5	25.4	0.004	0.06
#2	25.1	3.2	28.3	27.9	28.7	0.8	25.0	0.02	0.13
#3	25.2	5.1	30.3	29.4	30.5	1.1	21.5	0.04	0.19

**Table 3:** EDP and OET selected by SNR of temperature thermographic images.

Specimen	Test No.	Test Condition (Heating/Cooling, s)	Max. Contrast (°C)	EDP (Frames)	OET (Frame)
A-type	#1	10/40	1.82	50~150	70
	#2	20/80	2.48	50~240	80
	#3	30/120	2.76	60~350	130
B-type	#1	10/40	5.64	30~140	50
	#2	20/80	6.71	30~220	90
	#3	30/120	7.00	30~330	60

**Table 4:** EDP and OET selected by SNR of amplitude.

Specimen	Test No.	Test Condition (Heating/Cooling, s)	EDP (Frames)	OET (Frame)
A-type	#1	10/40	30~40	30
	#2	20/80	30~50	50
	#3	30/120	50, 90	50
B-type	#1	10/40	30~50	30
	#2	20/80	40~50	40
	#3	30/120	50, 100	50

**Table 5:** EDP and OET selected by SNR of phase.

Specimen	Test No.	Test Condition (Heating/Cooling, s)	EDP (Frames)	OET (Frame)
A-type	#1	10/40	20~30	20
	#2	20/80	20~30	20
	#3	30/120	20~40	30
B-type	#1	10/40	30	30
	#2	20/80	30~50, 80	30
	#3	30/120	30~50, 80~90	30

**Table 6:** Evaluated defect sizing and depth and error rate.

Type	Defect ID	Indicated Pixel Size ^(1)^	Evaluated Dia. (mm) ^(2)^	Real Dia. (Φ, mm)	Φ Error Rate (%)	SNR (dB) ^(3)^	Evaluated Depth (mm)	Real Depth (mm)	Error Rate (%)
A-type	A1	74	34.8	30	16	21.3	2.0	1.8	14
	A2	89	41.8	40	5	23.7	2.2	1.8	21
	A3	100	47.0	50	−6	26.4	2.4	1.8	35
	A4	65	30.6	30	2	28.1	3.3	3.0	11
	A5	83	39.0	40	−2	31.2	3.5	3.0	18
	A6	96	45.1	50	−10	33.2	3.7	3.0	22
	A7	57	26.8	30	−11	33.1	4.3	4.2	2
	A8	74	34.8	40	−13	36.7	4.6	4.2	9
	A9	89	41.8	50	−16	39.0	4.7	4.2	12
B-type	B1	95	44.7	40	12	9.2	0.5	0.6	−10
	B2	94	44.2	40	10	16.1	1.1	1.2	−12
	B3	81	38.1	40	−5	19.5	1.7	1.8	−7
	B4	82	38.5	40	−4	22.8	2.2	2.4	−10
	B5	78	36.7	40	−8	27.0	2.9	3.0	−3
	B6	74	34.8	40	−13	29.1	3.3	3.6	−7
	B7	72	33.8	40	−15	32.1	3.9	4.2	−8
	B8	70	32.9	40	−18	36.8	4.7	4.8	−3
	B9	65	30.6	40	−24	42.7	5.5	5.4	1

Note: ^(1)^ Number of pixels between the left and right sides of the binary image of the defective area. ^(2)^ Size calculated from pixel count and IFOV (in this study, 0.47 mm/pixel). ^(3)^ SNR calculated from the average temperature of the sound and each defect.

## Data Availability

The data are only available upon reasonable request.

## Abbreviations

The following abbreviations are used in this manuscript:

ATC	Absolute Temperature Compensation
ATT	Active Thermographic Testing
ECT	Eddy Current Testing
EDP	Easy Detectable Period
FFT	Fast Fourier Transform
FPA	Focal Plane Array
HA	Halogen Array
IR	InfraRed
LIT	Lock-In Thermography
LPT	Long Pulse Thermography
NDT	Non-Destructive Testing
NUC	Non-Uniformity Correction
OET	Optimal Evaluation Time
ORG	Original
PC	Personal Computer
PPT	Pulse Phase Thermography
PT	Pulsed Thermography
RT	Radiographic Testing
SHT	Step Heating Thermography
SNR	Signal-to-Noise Ratio
TRC	Temperature Rate Compensation
TSR	Thermographic Signal Reconstruction
TT	Thermographic Testing
UT	Ultrasonic Testing

## References

[1] Dwivedi S.K., Vishwakarma M., Soni A. (2018). Advances and Researches on Non Destructive Testing: A Review. Mater. Today Proc..

[2] Ida N., Meyendorf N. (2019). Handbook of Advanced Nondestructive Evaluation.

[3] Ibarra-Castanedo C., Maldague X.P. (2013). Infrared thermography. Handbook of Technical Diagnostics: Fundamentals and Application to Structures and Systems.

[4] Wang Z., Tian G., Meo M., Ciampa F. (2018). Image Processing Based Quantitative Damage Evaluation in Composites with Long Pulse Thermography. NDT E Int..

[5] Kim C., Kang S., Chung Y., Kim O., Kim W. (2023). Quantification of the Effective Detectable Period for Concrete Voids of CLP by Lock-in Thermography. Appl. Sci..

[6] Lee S., Chung Y., Kim C., Shrestha R., Kim W. (2022). Thermographic Inspection of CLP Defects on the Subsurface Based on Binary Image. Int. J. Precis. Eng. Manuf..

[7] Ciampa F., Mahmoodi P., Pinto F., Meo M. (2018). Recent Advances in Active Infrared Thermography for Non-Destructive Testing of Aerospace Components. Sensors.

[8] Vavilov V., Burleigh D. (2020). Infrared Thermography and Thermal Nondestructive Testing.

[9] Khodayar F., Sojasi S., Maldague X. (2016). Infrared Thermography and NDT: 2050 Horizon. Quant. InfraRed Thermogr. J..

[10] Chung Y., Lee S., Kim W. (2021). Latest Advances in Common Signal Processing of Pulsed Thermography for Enhanced Detectability: A Review. Appl. Sci..

[11] Yang R., He Y. (2016). Optically and Non-Optically Excited Thermography for Composites: A Review. Infrared Phys. Technol..

[12] Wang Z., Zhu J., Tian G., Ciampa F. (2019). Comparative Analysis of Eddy Current Pulsed Thermography and Long Pulse Thermography for Damage Detection in Metals and Composites. NDT E Int..

[13] Almond D.P., Angioni S.L., Pickering S.G. (2017). Long Pulse Excitation Thermographic Non-Destructive Evaluation. NDT E Int..

[14] Ding L., Ye Y., Ye C., Luo Y., He H., Zhang D., Su Z. (2023). Fourier Phase Analysis Combined with a Fusion Scheme in Long Pulse Thermography. Infrared Phys. Technol..

[15] Wei Y., Xiao Y., Gu X., Ren J., Zhang Y., Zhang D., Chen Y., Li H., Li S. (2024). Inspection of Defects in Composite Structures using Long Pulse Thermography and Shearography. Heliyon.

[16] Moskovchenko A., Švantner M., Honner M. (2024). Detection of Gunshot Residue by Flash-Pulse and Long-Pulse Infrared Thermography. Infrared Phys. Technol..

[17] Gomathi R., Ramkumar K. (2023). Defect Size Characterization in Unidirectional Curved GFRP Composite by TSR Processed Pulse and Lock in Thermography: A Comparison Study. J. Manuf. Eng..

[18] Miao Z., Wu D., Gao Y., Wang Y. (2023). Improved Long Pulse Excitation Infrared Nondestructive Testing Evaluation. Opt. Express.

[19] Cheng X., Chen P., Wu Z., Cech M., Ying Z., Hu X. (2023). Automatic Detection of CFRP Subsurface Defects Via Thermal Signals in Long Pulse and Lock-in Thermography. IEEE Trans. Instrum. Meas..

[20] Anwar M., Mustapha F., Abdullah M.N., Mustapha M., Sallih N., Ahmad A., Mat Daud S.Z. (2024). Defect Detection of GFRP Composites through Long Pulse Thermography using an Uncooled Microbolometer Infrared Camera. Sensors.

[21] Wei Y., Xiao Y., Li S., Gu X., Zhang D., Li H., Chen Y. (2024). Depth Prediction of GFRP Composite using Long Pulse Thermography. Measurement.

[22] Hyun H., Choi B. (2018). A Improved Scene Based Non-Uniformity Correction Algorithm for Infrared Camera. J. Korea Soc. Comput. Inf..

[23] Kim C., Kang S., Chung Y., Kim O., Kim W. (2024). Enhancement Contrast of Thermograms through Compensating for Uneven Irradiation in Thermographic Testing for Containment Liner Plates. J. Korean Soc. Nondestruct. Test..

[24] Vavilov V. (2009). Thermal/Infrared Testing. Nondestruct. Test. Handb..

[25] Choi M., Kang K., Park J., Kim W., Kim K. (2008). Quantitative Determination of a Subsurface Defect of Reference Specimen by Lock-in Infrared Thermography. NDT E Int..

[26] Maldague X., Moore P.O. (2001). Infrared and Thermal Testing.

[27] Kim C., Chung Y., Kim W. (2020). Optimization Method for evaluating Essential Factors of Plate Backside Thinning Defects using Lock-in Thermography. J. Korean Soc. Nondestruct. Test..

[28] Versaci M., Laganà F., Morabito F.C., Palumbo A., Angiulli G. (2024). Adaptation of an Eddy Current Model for Characterizing Subsurface Defects in CFRP Plates using FEM Analysis Based on Energy Functional. Mathematics.

